# Identifying disease phenotypes from linked health data: Comparison of self-report, hospital inpatient and primary care in UK Biobank.

**DOI:** 10.23889/ijpds.v9i5.1647

**Published:** 2024-09-18

**Authors:** Megan Conroy, Ben Lacey, Gillian Reeves, Naomi Allen

**Affiliations:** 1 University of Oxford

## Objectives

Administrative health data is commonly used for epidemiological research, however it is not well understood how disease phenotypes replicate across different data sources.

## Approach

UK Biobank is a prospective cohort study of 500,000 adults, with ascertainment of health outcomes using administrative health data. Prevalence at recruitment for 33 diseases were calculated in each health data source: self-report, primary-care, and hospital episode statistics (HES). Consistency of disease identification between sources, and median days between first diagnosis across data sources was determined. Linear regression was used to investigate determinant of differences in the average time between first diagnosis in primary-care and HES data.

## Results

3Hypertension was the most commonly identified disease in both self-report and HES (26.5% and 12.1% respectively), and anxiety in primary-care (12.7%). Diseases could be grouped into: 1) those identified largely by self-report alone (e.g. migraine, constipation), with inconsistency in the date first diagnosed; 2) those identified largely by primary care alone (e.g. anxiety, depression), also with inconsistency in the date first diagnosed; and 3) those that appeared mostly in all three sources, with highly consistent date of first report (many were emergency hospital admissions [e.g. stroke]). A number of variables were associated with time between primary-care and HES diagnosis. For example, heavier smokers had a significantly shorter period between first primary-care and first HES record for asthma, diabetes and hypertension.

## Conclusions and Implications

These results indicate that there are inherent biases in diseases ascertained from linked health data that must be taken into account for epidemiological studies.

